# Maintain, not obtain: rethinking HIV care in sub-Saharan Africa in the era of dolutegravir

**DOI:** 10.3389/fpubh.2026.1819844

**Published:** 2026-05-20

**Authors:** Carlo Federico Perno, Stefano Orlando, Noorjehan Abdul Majid, Hawa Sangare, Richard Luanga, Michele Roccucci, Loredana Andreea Silaghi, Zita Sidumo, Davide Brambilla, Susanna Ceffa, Giovanni Guidotti, Fausto Ciccacci

**Affiliations:** 1 Bambino Gesu Pediatric Hospital IRCCS, Rome, Italy; 2 Department of Biomedicine and Prevention, University of Rome Tor Vergata, Rome, Italy; 3 DREAM Program, Community of Sant’Egidio, Maputo, Mozambique; 4 DREAM Program, Community of Sant’Egidio, Blantyre, Malawi; 5 DREAM Program, Community of Sant’Egidio, Rome, Italy; 6 ASL Rome 1, Rome, Italy

**Keywords:** antiretroviral therapy, dolutegravir, drug-resistance, HIV care, viral suppression

## Abstract

Over the past four decades, the HIV epidemic in sub-Saharan Africa has shifted from an acute, high-mortality phase to an era of widespread antiretroviral therapy (ART), driven by global scale up and the introduction of potent regimens such as dolutegravir-based combinations. While viral suppression is now increasingly achievable, the central challenge has evolved toward maintaining durable virological control across the life course. We argue that program success can no longer be defined by the mere attainment of suppression, but by the sustained maintenance of undetectable viral load and preservation of drug effectiveness at the population level. From a virological perspective, incomplete suppression, including persistent low-level viremia, creates predictable conditions for resistance selection over time, even with high–genetic barrier agents. In settings where CD4 monitoring is infrequent, gradual immune deterioration may go unnoticed despite apparently acceptable viral load thresholds. Emerging integrase inhibitor resistance should therefore be interpreted as a systems-level warning signal rather than an isolated clinical event. We discuss dolutegravir as an extraordinary yet finite therapeutic resource, the risks of functional monotherapy when nucleoside backbones are compromised, and the role of postfailure strategies, including protease inhibitor–anchored regimens, within optimized care pathways. Central to this approach is recognizing diagnostics—viral load monitoring, CD4 assessment, and targeted genotypic resistance testing—as essential clinical infrastructure. Sustaining ART gains in Africa requires moving from “minimum for all” toward differentiated, high-quality lifelong care integrated within broader public health platforms.

## 1 Introduction

Over four decades, the HIV epidemic in sub-Saharan Africa has moved from an acute phase characterized by high mortality and limited therapeutic options to an era of large-scale coverage of antiretroviral therapy (ART) shaped by global financing mechanisms and programmatic goals such as 95–95–95 (1). This expansion has been further accelerated by the rapid rollout of dolutegravir (DTG)-based regimens across low- and middle-income countries. In a 35-country survey, 90% of HIV clinics had implemented DTG-based first-line regimens, while approximately 60–70% of patients on ART were transitioned to DTG within 2–3 years of rollout in sub-Saharan Africa (2–4). This transition has fundamentally altered both the natural history of infection and the demographic profile of people living with HIV; according to UNAIDS, AIDS-related deaths have declined from 2.1 million in 2004 to 630,000 in 2024. In the same period, the number of individuals requiring continuous care has grown, with people living with HIV increasing from 29.6 million to 40.8 million (1, 5, 6). The public health challenge is therefore no longer defined primarily by access to first-line therapy, but by the ability to maintain lifelong viral control, prevent treatment failure, and protect the effectiveness of key drug classes at the population scale (7). At present, neither a biological nor a functional cure is achievable, and an effective vaccine remains unavailable. HIV infection therefore requires lifelong treatment. This shift is especially consequential in Africa, where the age structure of the epidemic, the large cohort of children and young adults living with HIV, and the limited availability of advanced therapeutic alternatives make durability and surveillance central to equity and sustainability.

## 2 “Maintain, not obtain”: a lifetime virological endpoint

International treatment guidelines consistently emphasize durable viral suppression as the ultimate therapeutic goal, explicitly linking sustained undetectable viremia to reduced morbidity, mortality, and transmission risk (8–10). This concept is not merely programmatic but biologically grounded: prolonged low-level replication under antiretroviral pressure provides the substrate for resistance selection, even in regimens with a high genetic barrier. Consequently, viral suppression must be conceptualized as a dynamic, lifelong process rather than a binary or time-limited outcome.

From a virological perspective, applying this framework highlights persistent double standards in HIV care. In high-income settings, treatment success is defined by strict virological thresholds supported by regular monitoring. In contrast, in many African programs, lower expectations are often implicitly accepted due to resource constraints (11). This discrepancy risks normalizing suboptimal control and, over time, undermining treatment effectiveness at the population level. Recognizing heterogeneity across African countries is essential, but it should not translate into accepting inferior clinical endpoints. Rather, it calls for context-sensitive approaches that remain anchored to a common virological objective: sustained undetectable viral load throughout the patient’s lifetime (12).

## 3 The virology of resistance and the grey zone of low-level viremia

From a virological perspective, antiretroviral drug resistance is not an exceptional event but a predictable biological outcome when viral replication persists under pharmacological pressure. HIV exists as a dynamic swarm of quasispecies, driven by the error-prone activity of reverse transcriptase and by host cellular factors, including APOBEC-mediated editing. When antiretroviral therapy does not fully suppress replication, even at low levels, selective pressure favors variants carrying resistance-associated mutations, progressively reshaping the viral population toward reduced drug susceptibility. This process is cumulative and time-dependent: the longer replication continues in the presence of suboptimal drug pressure, the greater the probability that resistant variants will emerge and become dominant (13–15).

Insufficient drug potency, including functional potency, may result from subtherapeutic drug levels. These may in turn reflect intermittent adherence, pharmacokinetic variability, or drug–drug interactions. Pre-existing resistance, whether transmitted or selected during prior regimens, could further narrow the effective barrier to resistance, particularly when patients are switched to new combinations without full virological suppression. In addition, complex treatment histories with multiple prior failures could create viral populations that are already partially adapted to antiretroviral pressure (16, 17). In such contexts, resistance should be expected rather than considered a rare complication. The key virological message is therefore that resistance reflects system-level and treatment-level optimization: when therapy is not adequately tailored, monitored, and supported, resistance is the biologically logical endpoint rather than an unexpected accident. The abovementioned situation may be common in Africa, where millions of HIV-infected persons have been treated with first line regimens characterized by limited potency and/or low genetic barrier, such as those based upon first generation NRTI like AZT or d4T, together with first generation NNRTI such as nevirapine or efavirenz (third drug being usually 3TC). Under these conditions, in the absence of an adequate monitoring of viremia, it is more than conceivable that a substantial number of them failed therapy, developing resistance to NRTI and NNRTI (18). Highly potent regimens with a high genetic barrier, such as TLD (tenofovir + lamivudine + dolutegravir), may be less effective when used after failure of first-line therapies than when used in true first-line settings, where all three drugs are fully active. This may occur because resistance to NRTIs acquired during failure of earlier regimens can reduce or abolish the activity of the NRTI backbone, including tenofovir and lamivudine. In such cases, dolutegravir may become the only fully active drug in the regimen, resulting in so-called functional monotherapy. Under these conditions, TLD may fail, particularly in the presence of suboptimal adherence and rising viremia. This may ultimately lead to the emergence of dolutegravir resistance and make it much more difficult to identify a subsequent regimen with sufficient and durable efficacy, as recently highlighted in the context of DTG failure management (19). The consequences of this situation is further discussed below. Low-level viremia (LLV) represents a critical grey zone between apparent programmatic success and true virological failure. From a laboratory standpoint, viral load measurements are subject to analytical variability, particularly near the lower limits of detection, and programmatic cut-offs have historically been defined as pragmatic thresholds rather than biologically neutral boundaries. However, accumulating evidence indicates that persistent viremia in the range of 50–1,000 copies/mL is not virologically benign. Within this range, HIV replication can continue, maintaining selective pressure on viral populations and enabling the stepwise accumulation of resistance-associated mutations (20, 21).

Clinical and programmatic studies have shown that individuals with sustained LLV are at increased risk of subsequent virological failure and resistance emergence compared with those who achieve and maintain full suppression (22–24). From a public health perspective, accepting LLV as a satisfactory outcome may institutionalize a form of slow virological failure. Patients with viremia between 50 and 1,000 copies/mL may remain classified as “suppressed” while resistant variants are silently selected. Over time, this may lead to overt failure, onward transmission of resistant virus, and loss of therapeutic options at the population level. For these reasons, an undetectable-only standard should be considered the appropriate virological endpoint whenever possible, as it is the only acceptable standard of developed countries (25).

Programmatic thresholds and monitoring strategies must, therefore, be aligned with virological principles and with the diagnostic capacity of health systems, rather than being used to mask gaps in monitoring or access to care.

## 4 DTG as an extraordinary (yet finite) opportunity

The fixed-dose combination of tenofovir, lamivudine, and dolutegravir (TLD) has rightly been described as a programmatic “game changer,” enabling rapid scale-up of potent first-line ART with tolerability and virological efficacy comparable to regimens used in high-income settings (26–28). Yet, the very success of dolutegravir-based first-line therapy has created a new strategic challenge: in many low- and middle-income countries (LMICs), realistic, scalable “breakthrough” alternatives beyond DTG are limited, making DTG effectiveness a form of therapeutic capital that must be actively preserved. From a virological standpoint, the key vulnerability of DTG programs is not the intrinsic potency of DTG, but the context in which DTG is deployed (particularly among individuals with prior treatment failure and accumulated NRTI resistance). In such patients, recycling a compromised NRTI backbone (even changing the NRTI components, such as from the old AZT/D4T to tenofovir) can result in functional DTG monotherapy, a scenario that increases the probability of selecting integrase resistance mutations under persistent replication (29, 30). In this context, recent WHO recommendations have reinforced the role of protease inhibitor (PI)-based strategies after failure on DTG-containing regimens, emphasizing the use of a high–genetic barrier PI as the pharmacological “anchor” in subsequent lines of therapy; darunavir/ritonavir (DRV/r) is highlighted as the preferred PI option given its barrier to resistance and overall performance profile (31). Finally, the bias of functional monotherapy described from DTG after NRTI failures will apply also to darunavir (the most effective PI), whenever darunavir is used as the only effective drugs: after a beneficial effect driven by its intrinsic potency, there is a substantial risk that, over the following months, the virus replicates, develops resistance also to darunavir (and other PIs), ultimately causing a virological failure without further therapeutic options. This is particularly true in all cases when the next therapeutic regimen (either DTG- or PI-based) is provided without diagnostic procedures, but only based upon empirical knowledge. For a public health strategy, the core message is therefore not that PIs can substitute for diagnostics, but that PIs may be one component of a broader, system-oriented response. Without laboratory capacity (at minimum viral load), adherence support, and clear clinical pathways, a PI anchor risks becoming a costly patch rather than a sustainable solution over the years (always remembering that the therapy must be provided and be effective for decades, probably lifelong).

## 5 Emerging INSTI resistance

Multiple lines of evidence indicate that integrase strand transfer inhibitor (INSTI) resistance is an emerging and foreseeable challenge in sub-Saharan Africa (32). First, surveys have documented non-zero levels of pre-treatment INSTI resistance in several LMIC settings, challenging the assumption that INSTI resistance is negligible in regions scaling up DTG (33). Second, cohort and programmatic studies increasingly report acquired INSTI resistance among people experiencing virological failure on DTG-based regimens, including in settings where routine resistance testing is limited (30, 34). Scoping syntheses further suggest that emergent DTG resistance is heterogeneous across settings, but cannot be considered exceptional and may increase as DTG exposure accumulates (35, 36). The determinants of INSTI resistance in African programs are largely consistent with established virological principles. Persistent viremia at the time of regimen switching, particularly when a compromised NRTI backbone is recycled, creates conditions for functional DTG monotherapy and the selection of integrase mutations. Complex treatment histories and prior failures increase the likelihood of archived resistance, while intermittent adherence and models of service delivery that reduce clinical oversight can prolong periods of incomplete suppression. These risks are not theoretical: modeling work suggests that, in the absence of systematic monitoring and optimized failure management, the prevalence of acquired DTG resistance among individuals with virological failure could rise substantially over time, with important downstream consequences for program sustainability (37). Real-world data from Mozambique provide a concrete programmatic illustration of this trajectory. We documented that, in cohorts of individuals failing DTG-based regimens, almost half of the patients tested (46.4%) exhibit INSTI resistance mutations, often with recurring patterns such as G118R and R263K, sometimes accompanied by accessory mutations (e.g., L74M, E138K) that can further compromise susceptibility and limit future options (38, 39). It should be noted that the resistance to DTG nearly always hampers the future utilization of long-acting regimens based upon cabotegravir, an INSTI provided by a long half life, but devoided of a sufficient potency and genetic barrier to overcome the loss of efficacy driven by resistance mutations (40). Taken together, these findings are epidemiologically significant not only because they document resistance in routine care, but because they underscore the tight coupling between virological outcomes and health-system design: where diagnostics, adherence support, and timely clinical decisions are not consistently available, INSTI resistance becomes a predictable outcome rather than an anomaly.

## 6 The role of diagnostics in clinical management

If the goal of HIV care is lifetime virological control, diagnostics must be treated as core clinical infrastructure rather than an optional add-on. VL monitoring is the most direct marker of treatment effectiveness and the earliest signal of impending failure, while CD4 cell counts retain value for baseline risk stratification, advanced disease management, and clinical decision-making in settings where opportunistic infections remain common. All major guidelines consistently frame VL monitoring as central to ART management and recommend timely action when virological non-suppression is detected (9). Importantly, the case for “European-level” standards in African programs is not a rhetorical demand for identical systems, but a virological argument: the biology of resistance is universal, and programs that cannot detect and respond to replication are structurally predisposed to late failure and resistance amplification. Genotypic resistance testing (GRT) is often discussed in LMICs primarily as a surveillance tool, yet its highest value is clinical—guiding patient management at the moment when regimen decisions have long-term consequences as extensively reported in the abovementioned guidelines. The distinction matters: surveillance describes population patterns, whereas patient management requires actionable, timely results for an individual with detectable viremia. Correct timing is therefore critical. Resistance testing is most informative when performed while a patient is still taking the failing regimen (or shortly after discontinuation), before wild-type virus re-expands and masks resistant variants (41). This principle is reflected in guideline recommendations and in WHO’s HIV drug resistance strategy, which emphasizes the programmatic importance of virological monitoring linked to appropriate clinical responses (8, 18, 31). Operationally, implementing these standards in Africa is feasible through huband-spoke laboratory networks. In such models, peripheral clinics (“spokes”) collect specimens and ensure pre-analytical quality, while centralized or regional laboratories (“hubs”) perform VL and GRT with standardized workflows. Feasibility depends on logistics rather than conceptual novelty: specimen transport systems, quality assurance, defined turnaround times, and reliable result return to clinicians. The public health payoff is substantial because earlier detection and correct characterization of failure prevents prolonged replication under drug pressure. Economic considerations further strengthen the case. Empirical switching to protease inhibitor (PI)-based regimens without resistance information can be clinically effective in the short term. However, it may be inefficient at scale. It increases drug costs and toxicity burdens and does not address the underlying causes of non-suppression. Modeling and cost-effectiveness analyses suggest that targeted GRT in patients with persistent nonsuppression can produce both clinical and economic benefits by enabling rational switching decisions and avoiding unnecessary escalation to more complex regimens (42). In a lifetime-care paradigm, diagnostics should therefore be framed as an equity intervention: they prevent avoidable resistance, preserve future options, and reduce long-term costs (43). In programmatic HIV care, detectable viral load is not synonymous with drug resistance. Many episodes of non-suppression reflect intermittent adherence, treatment interruptions, or transient barriers to access. Treating all such cases as presumed resistance may lead to unnecessary GRT and premature switching to more complex or costly regimens that adherence support might have avoided. A sustainable approach therefore requires adherence-informed triage, supported where feasible by low-cost measures of drug exposure such as urine tenofovir testing and, in selected cases, plasma drug-level monitoring to identify those who should be prioritized for GRT and resistance-informed switching (44, 45). This same virology grounded logic should shape innovation. Long-acting antiretroviral therapy (LA-ART) with injectable cabotegravir plus rilpivirine (CAB/RPV) has the potential to reshape HIV service delivery by reducing daily pill burden and addressing structural, social, and psychological barriers to adherence. In African settings, where stigma, disclosure concerns, unstable access to care, and competing socioeconomic priorities may undermine consistent oral adherence, LA-ART could improve quality of life and retention in care, particularly for adolescents, highly mobile populations, and women facing social vulnerability (46). Early evidence from African implementation studies, including the CARES program, suggests that LA-ART can be feasible and acceptable in routine care under structured delivery conditions (47). Beyond currently available long-acting combinations, newer agents such as lenacapavir, a capsid inhibitor administered every 6 months, represent a further step in the evolution of long-acting antiretroviral therapy, with high rates of viral suppression demonstrated in clinical trials. However, their future implementation in low- and middle-income countries remains uncertain, due to cost, delivery constraints, and limited data in non-B subtypes and HIV-2 (48–50). From a virological perspective, however, the introduction of CAB/RPV in sub-Saharan Africa requires explicit guardrails mostly driven by the limited genetic barrier and potency of this association, both lower than other modern regimens such as TLD. First, rilpivirine vulnerability to pre-existing NNRTI resistance is a central concern in regions with long histories of NNRTI-based first-line therapy and incomplete virological monitoring. Undetected archived NNRTI resistance may predispose to early failure on CAB/RPV, particularly if eligibility is based on imperfect suppression histories. Second, the pharmacokinetic “tail”—a prolonged period of declining subtherapeutic drug levels after missed injections or discontinuation—creates a classical selection window for resistance. This is not merely a patient-level risk: at program scale, it can generate resistant variants that compromise future treatment options. A third concern is cross-resistance between cabotegravir and dolutegravir. Although dolutegravir has a higher genetic barrier, integrase resistance pathways often overlap. As a result, failure on a long-acting INSTI may compromise the effectiveness of DTG-based regimens, just as previous DTG failure may reduce the future utility of cabotegravir-based strategies in African programs (51). Modeling work and African-specific analyses have therefore emphasized that the predicted effectiveness of LA-ART is highly contingent on baseline resistance patterns and on systems that can ensure on-time dosing and early detection of failure (43). In practical terms, LA-ART should be positioned as an opportunity with conditions: careful patient selection, robust recall systems for injections, routine viral load monitoring, and access to genotypic resistance testing when clinically indicated. Without these safeguards, the public health risk is to trade short-term convenience for long-term loss of key drug classes. At the policy level, these considerations highlight the limits of “extreme simplification.” Service models that reduce clinical oversight, weaken adherence support, and delay virological monitoring can prolong incomplete suppression and promote resistance, ultimately turning manageable failure into programmatic fragility. Emerging INSTI resistance should therefore be interpreted as a systems-level signal rather than a rare clinical complication (52). The response is to treat guidelines as a floor rather than a ceiling and to pursue differentiated quality. This includes tailoring care pathways to country capacity while maintaining a common goal of durable undetectable viremia, strengthening diagnostics as core infrastructure, and integrating community-based adherence and retention support. The DREAM (Disease Relief through Excellence and Advanced Means) program illustrates that such models are feasible in resource-constrained settings (1, 53).

## 7 Conclusion

The scale-up of dolutegravir-based regimens represents one of the most transformative achievements in HIV control in sub-Saharan Africa. It has reshaped the therapeutic landscape and raised expectations for sustained virological control and reduced disease progression. At the same time, new steps are warranted; the next phase of the epidemic requires that this success be converted into durability: maintaining undetectable viral load over the life course is the keyword of new anti-HIV strategy, rather than merely achieving initial suppression. Emerging INSTI resistance should be interpreted as a warning signal of systemic weaknesses—namely incomplete monitoring, fragmented patient support, and delayed management of virological failure—all of which, cumulatively, threaten to undermine the very regimens that enabled rapid scale-up. While pragmatic strategies for managing failure—including PI-anchored approaches such as darunavir/ritonavir after DTG failure—may mitigate resistance risks, they cannot replace routine viral load monitoring, timely clinical action, and robust adherence support. Preserving DTG effectiveness therefore depends on health systems that minimize the duration of viral replication under drug pressure. Beyond HIV, the public health dividend of such investment is substantial: as survival improves, HIV clinics can function as platforms for integrated prevention and management of non-communicable diseases and for HBV and HPV vaccination, particularly benefiting women and children. The opportunity to move from “minimum for all” to “best possible for each” is immediate; postponing this shift risks entrenching late failure, rising resistance, and escalating costs at a moment when the therapeutic margin is narrowing.

## Data Availability

The original contributions presented in the study are included in the article/supplementary material, further inquiries can be directed to the corresponding author.
